# Mussel-inspired approach to constructing robust cobalt-embedded N-doped carbon nanosheet toward enhanced sulphate radical-based oxidation

**DOI:** 10.1038/srep33348

**Published:** 2016-09-12

**Authors:** Tao Zeng, Haiyan Zhang, Zhiqiao He, Jianmeng Chen, Shuang Song

**Affiliations:** 1 College of Environment, Zhejiang University of Technology, Hangzhou 310032, P. R. China

## Abstract

Heterogeneous sulphate radical based advanced oxidation processes (SR-AOPs) have lately been raised as a promising candidate for water treatment. Despite the progress made, either the stability or the performance of the current catalysts is still far from satisfactory for practical applications. Herein, using polydopamine-cobalt ion complex that inspired by mussel proteins as medium, we facilely fabricate a robust SR-AOPs catalyst with cobalt nanoparticles (NPs) embedded in nitrogen-doped reduced graphene oxide matrix (NRGO@Co). The NRGO scaffold with high porosity and surface area not only stabilizes the NPs but also greatly facilitates the accessibility and adsorption of substrates to the active sites. With the synergistic effect arising from the NRGO and Co NPs, the NRGO@Co hybrid catalyst exhibits enhanced catalytic activity for activation of peroxymonosulfate (PMS) to degrade organic pollutants in water. Furthermore, taking advantage of the favorable magnetic properties, the catalyst can be easily recycled and reused for at least 4 runs with negligible loss of activity. Coupled with systematic investigation in terms of influential factors, mineralization, and radicals identification, make the catalyst hold significant potential for application in remediation of organic pollutants in water.

Technological improvements in advanced oxidation processes (AOPs) are driven by an ever-increasing demand for removal of diverse pollutants emerged in water sources^1^,^2^,^3^. To avoid the precipitation of metal salts and difficulties in recycling, homogeneous reactions are progressively superseded by heterogeneous reactions^4^,^5^,^6^,^7^. To address the demerits of traditional hydroxyl radical (OH^**•**^), sulphate radical (SO_4_^•−^) with many virtues has lately been raised as a promising alternative for AOPs (namely, SR-AOPs)^8^,^9^,^10^,^11^. In this context, fabrications of heterogeneous catalysts for SR-AOPs spring up like mushrooms in recent years^12^,^13^,^14^. Despite the progress, there is still considerable room for improvement in the synthesis and properties of catalysts. On the one hand, it would be desirable to enhance the stability of the catalyst with simple and green method; this would minimize the harm to environment and the risk of activity decrease. On the other hand, replacing inert and limited-function catalyst supports (such as silica, Al_2_O_3_, and carbon material) with conducting and multifunctional ones would be highly beneficial for boosting the catalytic performance^15^,^16^,^17^. Thus, it is encouraging and important to develop more efficient and stable catalyst for SR-AOPs.

Recently, nitrogen-doped carbon materials, especially nitrogen-doped reduced graphene oxide (NRGO) that with unique two-dimensional structure and excellent electrical conductivity, have been recognized as superior candidate for catalyst support and catalytic material^18^,^19^,^20^,^21^,^22^. Apart from the widespread electrochemical applications^23^,^24^,^25^, several pioneer studies have shown that some nitrogen-doped carbon materials can also serve as metal-free SR-AOPs catalysts for pollutants remediation^26^,^27^,^28^,^29^. Thus, it is envisioned that utilizing NRGO sheets to support metal nanoparticles (NPs) as catayst for SR-AOPs would exhibit enhanced performance because that: (1) the highly conductive NRGO may facilitate the activation of oxidants in AOPs; (2) the graphitic carbon may offer adsorptive domains for enrichment of organic pollutants in proximity to catalysts^30^. However, producing such NRGO@NPs nanocomposites is not an easy task. Specifically, since the whole preparation procedure always makes up of GO-reducing, N-doping and NPs-loading segments, the available approaches are often tedious and ineluctably involve toxic reagents (such as N precursors, organic dispersing agents, linking molecules, and reductants)^31^,^32^,^33^,^34^. Another issue of concern is that the synthesis of active NPs-graphene hybrids is commonlly physically attaching NPs onto the surface of graphene with relatively weak interconnections, which could induce detachment and aggregation of NPs^35^.

Fortunately, dopamine, a biomolecule that contains amine and catechol groups, opens new opportunity for breaking through these limitations. Owing to self-polymerization feature, strong reducing ability and π-π stacking force with GO, dopamine can facilely integrate GO to form polydopamine (PDA)/RGO nanosheets^36^,^37^,^38^. In particular, latest reports found that catechol-ferric ion complexes in adhesive proteins greatly account for the prominent hardness and extensibility of mussel threads^39^. Inspired by these, it may be feasible to introduce metal ions into the dopamine polymerization for one-pot construction of robust metal NPs containing PDA/RGO materials. Thus, through carbonization, NRGO@NPs nanocatalyst can be obtained without the necessity of additional reagents or procedures.

Herein, using dopamine-cobalt ion complex as medium, we facilely fabricate N-doped graphitic layers supported Co NPs (NRGO@Co hybrids) with a regular morphology and demonstrate their application in serving as a novel SR-AOPs catalyst for pollutants removal. The NRGO scaffold with high porosity and surface area not only stabilizes the NPs but also greatly facilitates the accessibility and adsorption of substrates to the active sites. With the synergistic effect arising from the NRGO and Co NPs, the NRGO@Co hybrid catalyst exhibits highly efficient catalytic activity for activation of peroxymonosulfate (PMS) to degrade organic pollutants. Furthermore, long-term stability and magnetically recycling ability of the catalyst was also observed. Such hybrid nanomaterial therefore holds great promise in practical environmental remediation.

## Results and Discussion

### Synthesis and characterization

**Figure 1:**
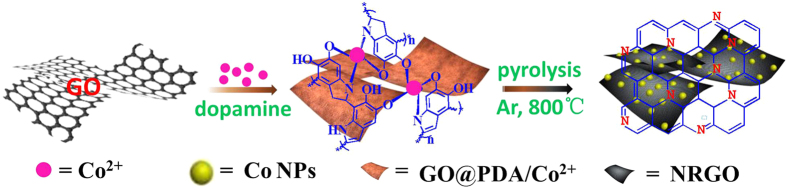
Schematic illustration (drawn by T. Z.) of the synthesis of NRGO@Co.

**Figure 2:**
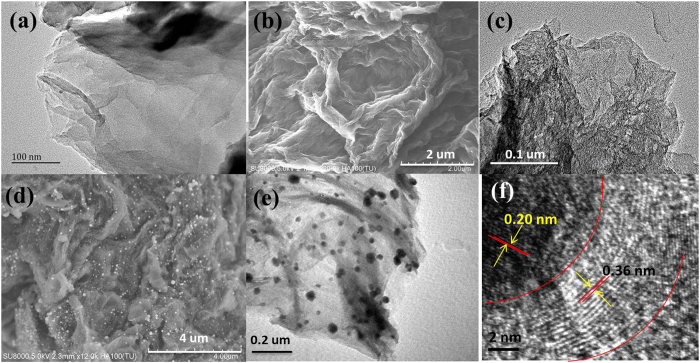
TEM image of GO@PDA/Co^2+^ (**a**) SEM (**b**) and TEM (**c**) images of NRGO; SEM (**d**) and TEM (**e**) images NRGO@Co; HRTEM image of core-shell structure of Co NP (**f**).

**Figure 3:**
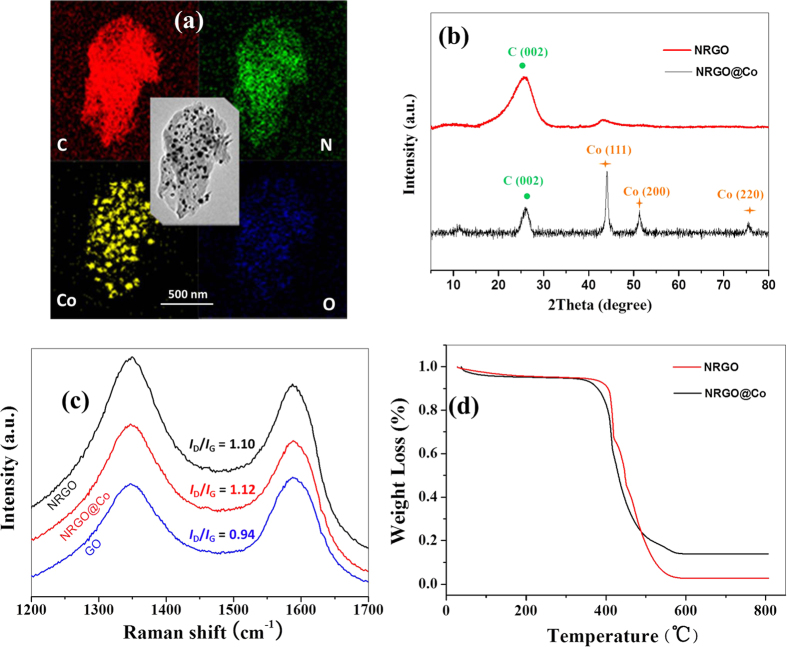
Elemental mapping of NNRGO@Co (**a**) XRD spectra (**b**), Raman spectra (**c**), and TGA spectra (**d**) of NRGO and NRGO@Co samples.

The synthesis route of NRGO@Co composite is illustrated in Fig. 1, which involves *in situ* polymerization of dopamine with Co species on GO surface followed by carbonization. In mixture, the complexation between PDA and Co ions together with π-π stacking between GO and reductive PDA give fairly stable solid products GO@PDA/Co^2+^. Upon annealing in Ar atmosphere, the GO/PDA was carbonized into NRGO whereas Co^2+^ was thermally reduced into Co NPs, which employs environmentally friendly N precursors of dopamine instead of toxic reagents like NH_3_ or ammonium nitrate. As displayed by the TEM image of GO@PDA/Co^2+^ (Fig. 2a), the complex is homogeneous and preserves the structural integrity of the parent GO sheets. The SEM and TEM images in Fig. 2d,e show that the homogeneous samples are converted into heterogeneous materials with a large amount of Co NPs uniformly embedded in the wrinkled carbon matrix after carbonization. The Co NPs with a size range of 20–38 nm are rarely aggregated, indicating a good dispersion in the carbon structure. High-resolution TEM (HRTEM) image focusing on a single Co NP (Fig. 2f) reveals distinct lattice fringes with a *d*-spacing of 0.20 nm, corresponding to the spacing between the (111) planes of nanocrystalline Co. Meanwhile, it can be observed that single Co NP is surrounded by a thin graphitized carbon shell from the HRTEM image. To identify the role of NRGO in AOPs, NRGO without Co species was also prepared. The SEM (Fig. 2b) and TEM image (Fig. 2c) of pure NRGO show a similar morphology to that of NRGO@Co hybrid except the vanishment of Co NPs. The elemental mapping for NRGO@Co hybrid further elucidates the co-existence and well-defined spatial distribution of C, N, Co, and a small amount of O species (Fig. 3a). Such a structure of small Co NPs uniformly embedded in N-doped carbon matrix could effectively keep the Co NPs stable and accessible, and give full scope to respective roles or synergistic effect of the two components, which would render the hybrids excellent in catalytic oxidation. X-ray diffraction (XRD) analysis (Fig. 3b) was also conducted to compare their crystalline structure. Apart from the diffraction hump at about 26.2° assigned to the (002) plane of graphitic carbon, the pattern of NRGO@Co hybrid presents three additional diffraction peaks at around 44.3°, 51.6°, and 75.8° that not available in the pattern of NRGO, which further confirming the presence of metallic Co in NRGO@Co composite.

The Raman spectra (Fig. 3c) of NRGO with/without Co NPs products all display well-documented D band at 1350 cm^−1^ and G band at 1580 cm^−1^, corresponding with the disordered graphitic carbon and the E2g vibration of the sp^2^-bonded carbon atoms, respectively, which are characteristics of graphitic-like materials, implying the generation of graphitic carbon during the pyrolysis. The extent of disorder degree and average size of the sp^2^ domains in graphite materials can be represented by the intensity ratio *I*_D_/*I*_G_. The values of *I*_D_/*I*_G_ were calculated as 1.10 for NRGO, 1.12 for NRGO@Co, and 0.94 for GO, respectively. The *I*_D_/*I*_G_ values of the pyrolysis products (NRGO and NRGO@Co) are higher than that of GO precursor, indicating the formation of more defects and disorders of graphitized structures due to the substitution of nitrogen atoms and the decrease in average size of the sp^2^ domains upon the generation of NPs on the graphene sheets. On the basis of the assumption that all Co has transferred to Co_3_O_4_ and all C has been burn out, thermogravimetric (TG) analysis (Fig. 3d) reveals that the mass fraction of Co in NRGO@Co is about 7.3 wt%.

**Figure 4:**
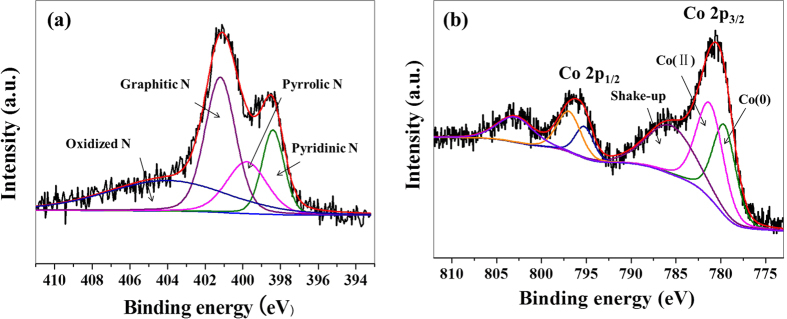
N 1s core-level spectrum of NRGO@Co catalyst (**a**), and Co 2p core-level spectrum of NRGO@Co catalyst (**b**).

X-ray photoelectron spectroscopy (XPS) was used to investigate the surface elemental composition of NRGO@Co hybrid. As shown in Fig. S1, a set of peaks in relation to C 1s, N 1s, O 1s, and Co 2p appears, which is in line with the elemental mapping results, providing a supportive evidence for successful N-doping and Co NPs formation. The N 1s core-level spectrum can be deconvoluted into four peaks corresponding to pyridinic N (398.4 eV), pyrrolic N (399.8 eV), graphitic N (401.2 eV), and oxidized N (404.0 eV) (Fig. 4a), and the percentage of graphitic species rank first. Some previous studies reported that graphitic N and pyridinic N, rather than pyrrolic N and oxidized N, are favorable to improve the electrochemical performance^40^,^41^, while other reports demonstrated that all of the pyridinic N, pyrrolic N, and graphitic N can boost oxygen reduction except oxidized N^42^,^43^. Anyway, the presence of pyridinic N, pyrrolic N, and graphitic N in N 1s spectrum implies that the as-prepared NRGO might have positive effect on the activity of the hybrid catalyst. The Co 2p core-level spectrum (Fig. 4b) are consist of Co 2p_3/2_ and Co 2p_1/2_ peaks, which are both fitted with three constituents in regard of Co(0), Co(II), and the shake-up peaks. The appearance of Co^2+^ oxides in the XPS analysis manifests that the Co NPs in NRGO@Co composite suffer from partial oxidation. This may be originated from the fact that exposure of Co NPs to ambient air could cause the formation of a thin CoO shell since the cobalt (0) nanoclusters are sensitive to aerobic atmosphere.

The porosity and surface area of the resulting materials were characterized by N_2_ adsorption−desorption measurements (Fig. S2). Calculated from the desorption branch of the isotherm with BJH method, the porosity and surface area of NRGO@Co composite are determined to be 0.55 cm^3^ g^−1^ and 568.3 m^2^ g^−1^, respectively, which are much higher than those of bare NRGO (0.19 cm^3^ g^−1^ and 188.8 m^2^ g^−1^). The changes in the porosity and surface area must be originated from the growth of Co NPs during the pyrolysis process. Such high porosity and surface area would facilitate the facile adsorption and diffusion of organic molecules into the NRGO@Co hybrid. To explore potential applications, it is very interesting to study the magnetic properties of NRGO@Co hybrid. Figure S3 illustrates its room-temperature magnetization curves, which exhibits a hysteresis loop, revealing ferromagnetic characteristic of the NRGO@Co hybrid. Saturation magnetization is measured to be 13.51 emu g^−1^ and typically comes from the large loading amount of Co NPs, endowing an easy and convenient way to magnetically separate NRGO@Co from the reaction system.

### Catalytic activity

**Figure 5:**
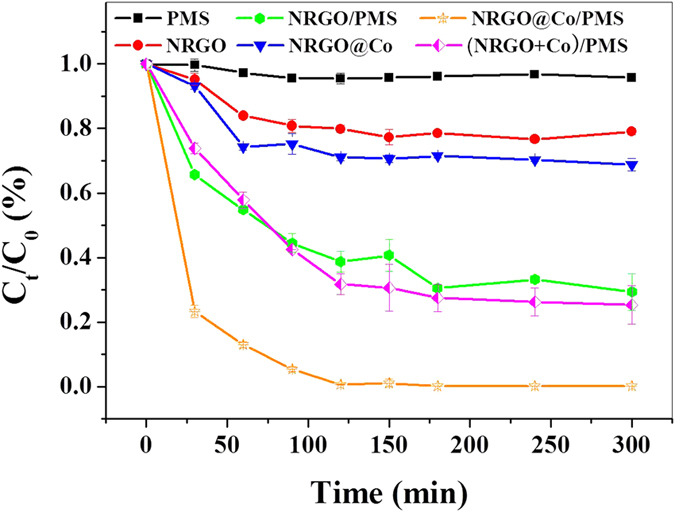
Removal of 4-CP under different situations (catalyst 0.2 g L^−1^, initial 4-CP 20 mg L^−1^, PMS 2 g L^−1^, temperature 298 k). Error bars stand for the standard deviation from the mean.

To investigate the catalytic performance of the as-prepared catalysts, oxidation degradation of 4-chlorophenol (4-CP) by activated peroxymonosulfate (PMS) was chosen as a model reaction, and a set of control experiments were conducted. The adsorption and degradation profiles of 4-CP against the reaction time in various conditions are shown in Fig. 5. PMS itself can scarcely generate active radicals for 4-CP degradation without a solid catalyst. Meanwhile, without PMS, both NRGO and NRGO@Co only showed a limited performance of 4-CP removal due to adsorption. NRGO@Co exhibited a superior adsorptive ability (about 29% of 4-CP) to NRGO material (about 22% of 4-CP), possibly on account of its higher porosity and surface area. NRGO showed a moderate activity in the presence of PMS under the given conditions and nearly 70% of 4-CP was removed, evidencing the NRGO support can individually activate PMS for decomposition of 4-CP. Sun *et al*. recenltly found N-doped nanocarbons possess of PMS activation ability for phenol degradation, which is in accordance with above result^44^. Notably, 4-CP could be almost completely degraded within 120 min using NRGO@Co as catalyst to activate PMS, implying a higher catalytic efficiency of it than those of other tested control material (Co_3_O_4_, Fe_3_O_4_ and NRGO) (Fig. S4). To verify whether the performance is caused by the additive effect of NRGO and Co NPs, catalytic degradation of 4-CP by mixed addition of equivalent pure NRGO and Co NPs to NRGO@Co hybrids was carried out. It can be seen that the mixture of pure NRGO and Co NPs showed just a little more than 70% of 4-CP removal, which is far from the catalytic performance of NRGO@Co hybrids, suggesting the catalytic performance cannot be simply ascribed to the additive effect of NRGO and Co NPs. According to pseudo-first-order reaction, the rate constants for NRGO and NRGO@Co were estimated to be 0.0076 and 0.0381 min^−1^, respectively. NRGO@Co presented 5-fold enhancement over NRGO catalyst, which may be originated from the synergistic effect arising from the Co NPs and NRGO supports. To distinguish the possible contribution of the leachate to the reaction, filtrate from solid catalyst suspension being shaken for a certain time was applied to catalyze the degradation of 4-CP. Little 4-CP removal was observed, indicating the degradation of 4-CP mainly occurs in a heterogeneous reaction rather than a homogeneous reaction.

Further tests were performed to evaluate the effect of reaction parameters. Figure S5a displays the impact of initial 4-CP concentration on its degradation. It was found that 4-CP removal efficiency tended to drop quickly with increasing initial 4-CP contents in solution. Complete removal of 4-CP needed 60 and 120 min for 10 and 20 mg L^−1^, respectively, whereas only 81% 4-CP was eliminated in 150 min at a concentration of 50 mg L^−1^. This may be caused by the coverage of excess 4-CP molecules and intermediates on the active sites of the catalyst. Moreover, the insufficient PMS might also be a limiting factor for the elimination of high concentration of 4-CP. Figure S5c shows the effect of catalyst dosage on 4-CP removal. It can be seen that high catalyst consumption would improve the 4-CP degradation significantly, and the removal efficiency of 4-CP in 30 min increased from 60% to 85% with the dosage of catalyst increasing from 0.1 to 0.5 g L^−1^. Higher dosage of catalyst will bring in more active sites to activate PMS, giving rise to the enhancement of catalytic efficiency.

**Figure 6:**
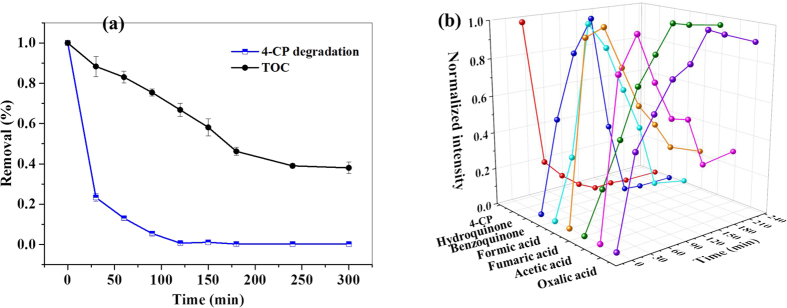
The efficiency of TOC removal during the degradation of 4-CP (**a**) and the evolution of the main intermediates with reaction time (**b**). Error bars stand for the standard deviation from the mean.

During the oxidation process, the TOC removal was measured to reflect the degree of oxidative destruction of 4-CP. Figure 6a discloses the mineralization profile of 4-CP with reaction time in the reaction system. As observed, the TOC reduction proceeded much more slowly than did the removal of 4-CP. The 4-CP removal was approximately 90% after 60 min, but the TOC was eliminated only less than 20%. It is speculated that the production of intermediates accompanying the oxidation makes the TOC can hardly be reduced as fast as 4-CP, and HPLC analysis verified hydroquinone and benzoquinone were the main benzenoid intermediates (Fig. 6b). Moreover, the IC detection results demonstrate that some smaller molecular organic acids including formic acid, fumaric acid, acetic acid, and oxalic acid were generated during the reaction, and eventually left in the reaction solution. The mineralization can eventually reach to 62% after 240 min, and the residual TOC may associate with those recalcitrant small molecular carbolic acids.

**Figure 7:**
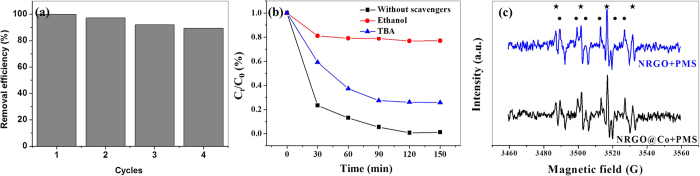
Reusability of the NRGO@Co catalyst in the degradation of 4-CP (**a**); effect of radical quenching on 4-CP degradation (**b**); EPR spectra of PMS activation with NRGO and NRGO@Co catalysts (**c**).

The reusability of the catalyst is a crucial concern with regard to the potential application. To assess it, the used catalyst was collected and reused in the next run with the same conditions. Taking advantage of the favorable magnetic properties, the catalyst can be easily separated from the reaction mixture and the cloudy suspended solution turns transparent within a few seconds with an external magnet. As shown in Fig. 7a, the NRGO@Co catalyst remained active with a removal efficiency of 4-CP nearly 90% within 120 min even after four successive rounds. The slight decrease of removal efficiency may be ascribed to the loss of recovered catalyst in each cycle. The change of surface chemistry of the hybrid before and after used was also evaluated by the XPS analyses of the elemental contents (Fig. S6). The fresh NRGO@Co hybrid is composed of C, N, O, and Co, with average contents of ~92.04 wt.%, ~2.49 wt.%, ~3.37 wt.% and ~2.10 wt.%, respectively. After reaction, the C content in NRGO@Co hybrid decreased by ~7.12 wt.%, whereas the content of O, Co is increased by ~5.61 wt.%, and ~1.43 wt.%, respectively. The decrease of C content may be originated from the long-term surface carbon etching by the reaction mixture, which in turn increases the exposure chance of the Co NPs, leading to a slight increase of Co content. In the case of the increase of O content, the surface hydroxylation of the hybrid in the Fenton-like degradation process might be responsible for this observation. Additionally, TEM and FTIR measurements were conducted to check the possible change in morphology ofNRGO@Co catalyst before and after use and the results showed no significant change (Fig. S7), indicating the catalyst remained very well. The good stability and reusability therefore render the NRGO@Co catalyst hold great promise for water treatment application.

### Possible reaction mechanism

Several radicals including OH^•^, SO_4_^•−^, and peroxymonosulfate radical (SO_5_^•−^) could be generated in heterogeneous activation of PMS^45^. Normally, OH^•^ and SO_4_^•−^ could attack the organic molecular while SO_5_^•−^ not due to its much lower redox potential. Ethanol and *tert*-butyl alcohol (TBA) by means of their different reaction rate with OH^•^ and SO_4_^•−^ (the reaction rate of TBA with OH^•^ is much higher than with SO_4_^•−^ while the reaction rate of ethanol with OH^•^ is comparable to SO_4_^•−^) were used for quenching experiments to check the radical type present in the reaction. Figure 7b depicts the inhibition effect of the two quenchers on the 4-CP degradation in NRGO@Co/PMS system. Addition of ethanol vastly inhibited the degradation of 4-CP, leading to the decrease of 4-CP removal to 20%. The presence of TBA resulted in 71% 4-CP degradation as compared to 100% 4-CP degradation without addition of alcohols. Thus, both OH^•^ and SO_4_^•−^ were suggested as the radical species in the studied system and SO_4_^•−^ was the primary one. Also, EPR was employed to straight probe the generation of radicals using 5,5-dimethyl-1-pyrroline (DMPO) as a radical spin trapping agent. Figure 7c demonstrates that both NRGO and NRGO@Co were able to effectively activate PMS to generate OH^•^ and SO_4_^•−^. The C 1s core-level is able to be fitted to four components at ~284.6, ~285.3, ~286.5, and ~290.1 eV, corresponding to C-C, C-OH, C-O-C, C=O groups, respectively (Fig. S8a,b). The proportion of C-OH in C 1s core-level spectra increased from 25.3% to 31.3% after the reaction provides a supportive evidence for the surface hydroxylation of the hybrid and the generation of OH^•^ in the degradation process.

**Figure 8:**
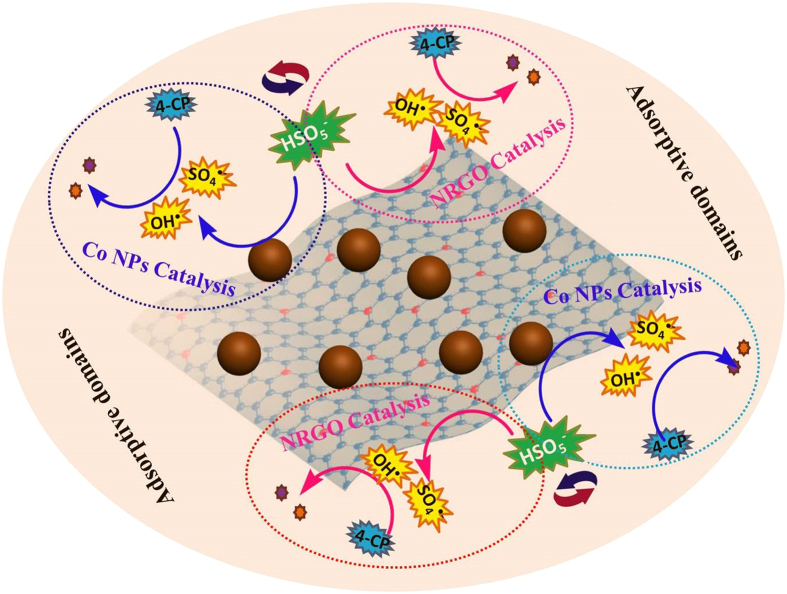
Possible mechanism of PMS activation on NRGO@Co to decompose 4-CP (drawn by T. Z.).

It was reported that the graphitic N with higher electronegativity and smaller covalent radius can facilitate the electron transfer from the adjacent C to N, thus altering the chemical nature of sp^2^ carbon layer and giving rise to appreciable catalytic activity of graphene^44^. Several groups also proved that the graphitic N sites have particular catalytic activity toward oxygen reduction reaction^25^. Accordingly, we propose that graphitic N in NRGO support make it hold chemical activity to activate PMS to produce radicals. XPS survey after catalyst used shows that the relative proportion of graphitic N in the N 1s core-level spectrum is changed (Fig. S8c,d), manifesting graphitic N did play a key role in PMS activation. Besides, considering that the cobalt with variable chemical valences is able to transfer electrons and serve as the active site, the Co NPs in nanocomposites could also be effective in activation of PMS. As for the Co 2p_3/2_ XPS spectra (Fig. S8e,f), the intensity ratio of Co (II) to Co (0) after NRGO@Co used slightly increased from 1.23 to 1.63, which confirms the oxidation of Co (0) to Co (II) species during the reaction between Co (0) and PMS. The high-density Co based sites in NRGO@Co are uniformly distributed all over the carbon matrix and are well accessible, rendering the nanocomposites high-efficiency in the catalytic system. Then, a possible mechanism of NRGO@Co in the oxidative degradation of 4-CP with PMS was proposed as follows (Fig. 8). First, the N-doped carbon matrix offers specific adsorptive domains for the enrichment of PMS and 4-CP from bulk solution through chemical adsorption and *π–π* interaction, respectively, leading to a high concentration of reactants around the nanocatalyst. Second, the oxidizing agent was catalytically activated by collective action of Co NPs and NRGO supports in the hybrid, creating abundant reactive species. Third, the adjacent 4-CP molecules were *in situ* oxidatively degraded to small molecular substances, entirely releasing the occupied sites of NRGO@Co as free sites.

In summary, a facile route has been developed to fabricate Co NPs uniformly embedded in NRGO matrix by one-pot pyrolysis of GO@Co^2+^/PDA ternary complex. In addition to effectively avoiding the aggregation of incorporated high-density NPs, the approach makes the resultant NRGO@Co catalyst inexpensive, stable, and magnetically recyclable. Benefiting from the special structure of the nanocomposite and the synergistic effect between Co NPs and NRGO support, the catalyst showed high-efficiency activity to activate PMS for oxidative degradation of 4-CP. Competitive radical tests and EPR analysis suggested that both OH^•^ and SO_4_^•−^ were generated during the activation processes and played key roles for 4-CP degradation. The negligible loss of 4-CP removal after four consecutive cycles indicated the NRGO@Co catalyst also has satisfactory reusability. Such findings highlight the potential of this robust and multifunctional catalyst as a realizable platform for efficient environmental remediation.

## Methods

### Chemicals

2-Amino-2-hydroxymethylpropane-1, 3-diol (Tris), 3-Hydroxytyramine hydrochloride (dopamine), and 4-chlorophenol (4-CP) were obtained from Acros Organics (Morris Plains, NJ). Co(NO_3_)_2_·6H_2_O, Tert-butyl alcohol (TBA) and ethanol were from Sinopharm Chemical Reagent Co. Ltd. (Shanghai, China). Peroxymonosulfate (PMS, 2KHSO_5_·KHSO_4_·K_2_SO_4_, Oxone) was supplied by Sigma-Aldrich. HPLC-grade methanol was supplied by Fisher Scientific (Fair Lawn, NJ). Graphene oxide (GO) was supplied by XFNano Materials Tech Co., Ltd. (Nanjing, China). All chemicals were used as received without any further purification. Ultrapure water was prepared in the laboratory using a Milli-Q SP reagent water system from Millipore (Milford, MA).

### Preparation of NRGO@Co hybrids

Aqueous suspension of graphene oxide (250 mL of a 0.2 mg mL^−1^ solution) was first prepared by 30 min sonication of GO. The pH of the solution was adjusted to 8.5 by using Tris buffer (10 mM). Then, Co(NO_3_)_2_·6H_2_O (1.3 mmoL) was dissolved in the mixture, followed by the addition of dopamine (2 mg mL^−1^). After stirring for 24 h, the GO@PDA/Co^2+^ complex was obtained from the solution by centrifugation and freeze-dried. Finally, NRGO@Co hybrids were prepared by annealing GO@PDA/Co^2+^ complex in a tube furnace at 800 °C for 3 h at argon atmosphere. NRGO material was synthesized through a similar procedure without the addition of cobalt salt.

### Catalytic activity test

All the experiments were conducted in a conical flask (50 mL) in the dark with a rotate speed of 300 rpm. The reactions were carried out in 4-CP aqueous solution (20 mg L^−1^) with a NRGO@Co catalyst (0.2 g L^−1^) and PMS (2 g L^−1^) at room temperature at neutral pH. Samples (0.2 mL) were taken out at a given time intervals and quenched with excess pure methanol (0.2 mL) and then centrifuged for the following analysis. Dionex ultimate 3000 HPLC (Dionex, Sunyvale, CA) with a PDA-100 photodiode array detector and an Acclaim 120 C18 column (5 um, 4.6 × 250 mm) was applied to analyze 4-CP and some intermediates. The mobile phase was composed of acetonitrile and water (70:30, v/v) at a flow rate of 1.0 mL min^−1^ with a column temperature of 30 °C. The detection wavelengths of 4-CP, hydroquinone, and benzoquinone were set at 280 nm, 290 nm, and 245 nm, respectively. TOC was measured by TOC/TN analyser (liquic TOCII) (Elementar Corporation, Germany) with deionized water and 0.8% HCl as mobile phase. For the recycle tests, the hybrids was recovered, washed with deionized water, dried and used in the next run with the similar experimental conditions.

### Characterization

The morphologies of the synthesized materials were surveyed by Tecnai G2 F20 HRTEM with an energy dispersive X-ray spectrometry (HRTEM-EDX, FEI, Netherlands), scan electronic microscopy (SEM, Hitachi SU8020, Japan). X-Ray diffraction studies (XRD, PANalytical X’ Pert diffractometer, Almelo, Netherlands) were performed by using a monochromatized X-ray beam with nickel-filtered Cu Kα radiation with 0.4° min^−1^ scan rate. X-Ray photoelectron spectroscopy (XPS) measurements were conducted by applying a Thermo Scientific ESCA-Lab-200i-XL spectrometer (Waltham, MA) with monochromatic Al Kα radiation (1486.6 eV). FTIR spectra were taken in KBr pressed pellets on a Nicolet Thermo NEXUS 670 Infrared Fourier Transform Spectrometer (Waltham, MA). Thermogravimetric analysis (TGA) was conducted on a Mettler Toledo TGA/DSC1 STAR^e^ system (Columbus, OH). Samples were loaded into a pan and heated to 800 °C at a rate of 10 °C/min. Nitrogen sorption isotherms were measured at 77 K with a Quadrasorb™ SI Four Station Surface Area Analyzer and Pore Size Analyzer (Quantachrome Instruments, Boynton Beach, FL). Before measurements, the samples were degassed in a vacuum at 300 °C for at least 6 h. The Brunauer-Emmett-Teller (BET) method was utilized to calculate the specific surface areas using adsorption data in a relative pressure range from 0.05 to 1.0. The pore volumes and pore size distributions were calculated by using the Barrett-Joyner-Halenda (BJH) model and the total pore volumes were estimated from the adsorbed amount at a relative pressure P/P_0_ of 0.994. An EMS-plus EPR instrument from Bruker was employed to detect the free radicals captured by 5,5-dimethyl-1-pyrroline (DMPO, >99.0%) during PMS activation, operating under the following conditions: center field, 3515 G; sweep width, 100 G; microwave frequency, 9.87 GHz; power setting, 18.75 mW; scan number, 3.

## Additional Information

**How to cite this article**: Zeng, T. *et al*. Mussel-inspired approach to constructing robust cobalt-embedded N-doped carbon nanosheet toward enhanced sulphate radical-based oxidation. *Sci. Rep.* **6**, 33348; doi: 10.1038/srep33348 (2016).

## Supplementary Material

Supplementary Information
