# Supplementary material for: Mussel-inspired approach to constructing robust cobalt-embedded N-doped carbon nanosheet toward enhanced sulphate radical-based oxidation
Source: Sci Rep. 2016 Sep 12;6:33348. doi: 10.1038/srep33348 (PMC5018839; doi:10.1038/srep33348)
Supplement: Supplementary Information [file srep33348-s1.pdf]

Mussel-inspired approach to constructing robust cobalt-embedded N-doped carbon nanosheet toward enhanced sulphate radical-based oxidation

Supplementary Information

Tao Zeng, Haiyan Zhang, Zhiqiao He, Jianmeng Chen and Shuang Song *

College of Environment, Zhejiang University of Technology, Hangzhou 310032, P. R. China. Email: ss@zjut.edu.cn; Fax: +86-571-88320276; Tel: +86-571-88320726

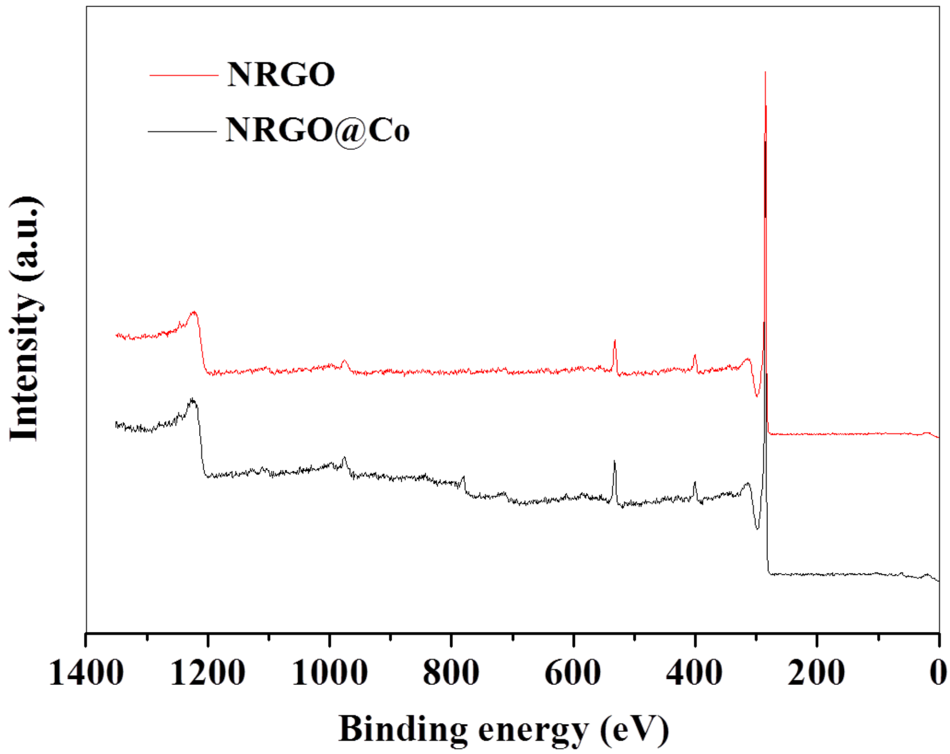

Figure S1. XPS wide scan survey of samples.

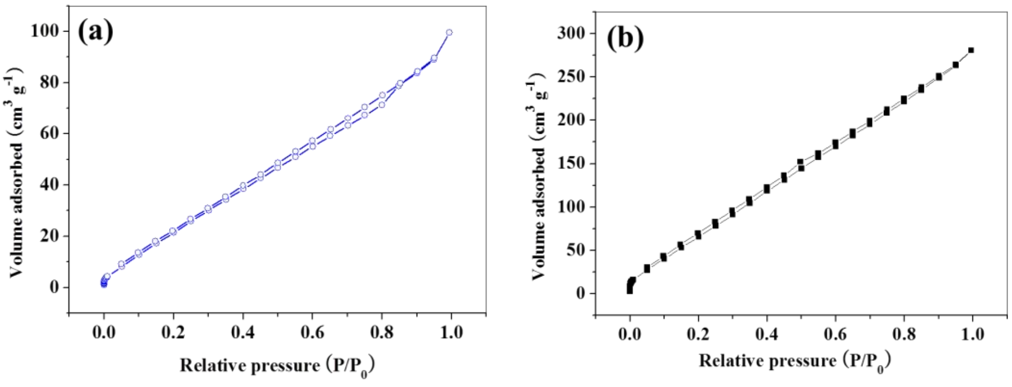

Figure S2. N_2 adsorption−desorption spectra of NRGO (a) and NRGO@Co (b).

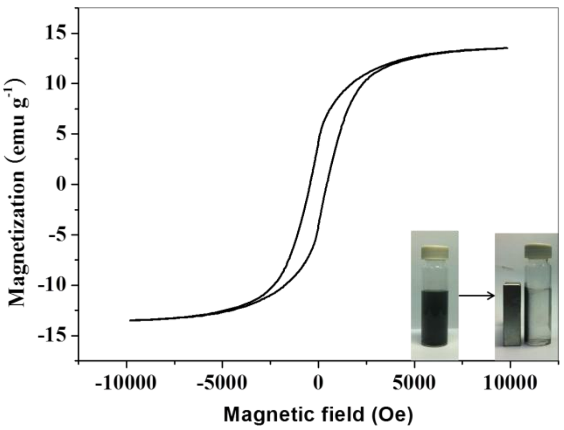

Figure S3. Magnetization curves of NRGO@Co hybrid. The inset pictures (taken by T.Z.) show the magnetic separation of the catalyst in mixture.

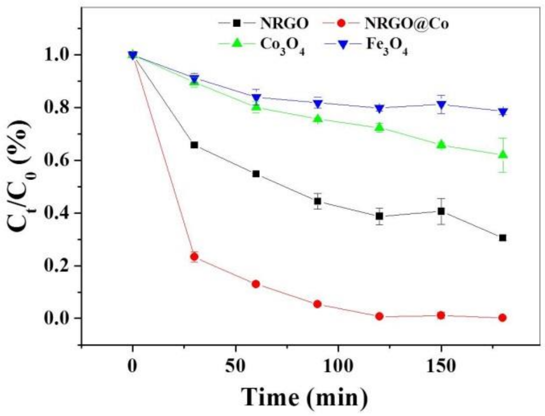

Figure S4. Comparison of the catalytic performance of various SR-AOPs catalysts. (catalyst 0.2 g L^{-1}, initial 4-CP 20 mg L^{-1}, PMS 2 g L^{-1}, temperature 298 k).

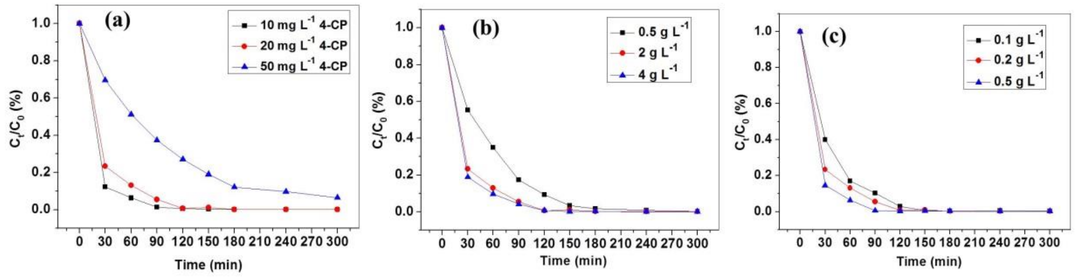

Figure S5. Effects of initial 4-CP concentration (a), PMS concentration (b), and catalyst loading (c) on 4-CP degradation.

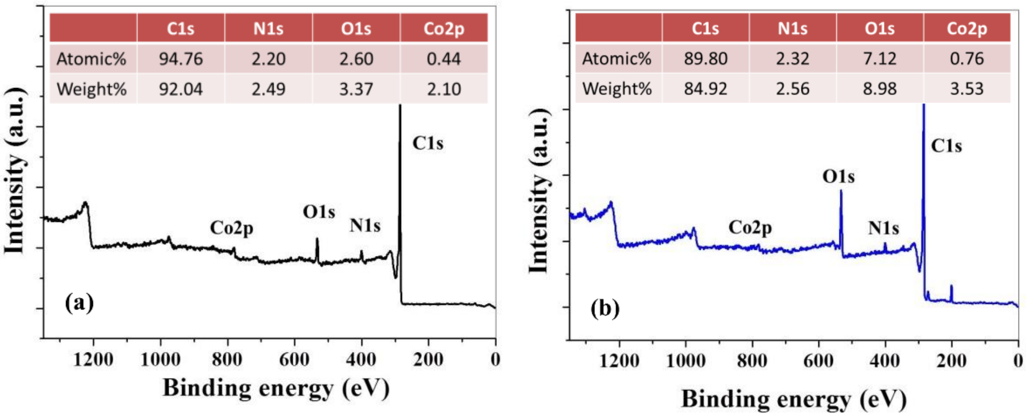

Figure S6. XPS wide scan survey and elemental contents of NRGO@Co hybrid before (a) and after used (b).

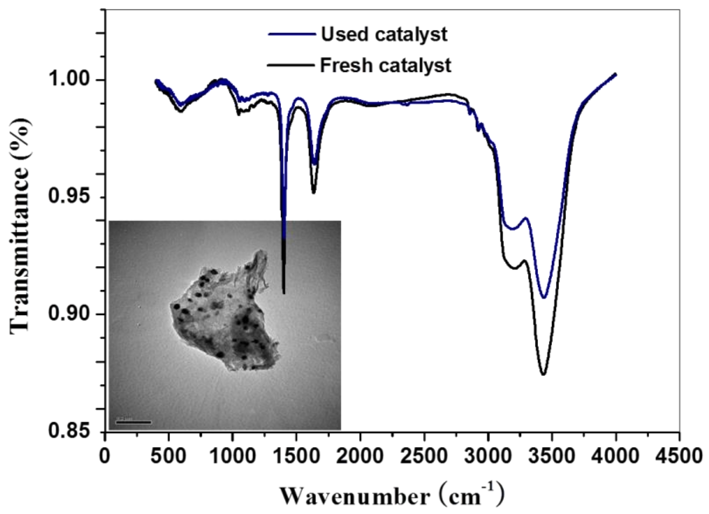

Figure S7. FTIR spectra of catalyst before and after use. The inset is the TEM image of catalyst after use.

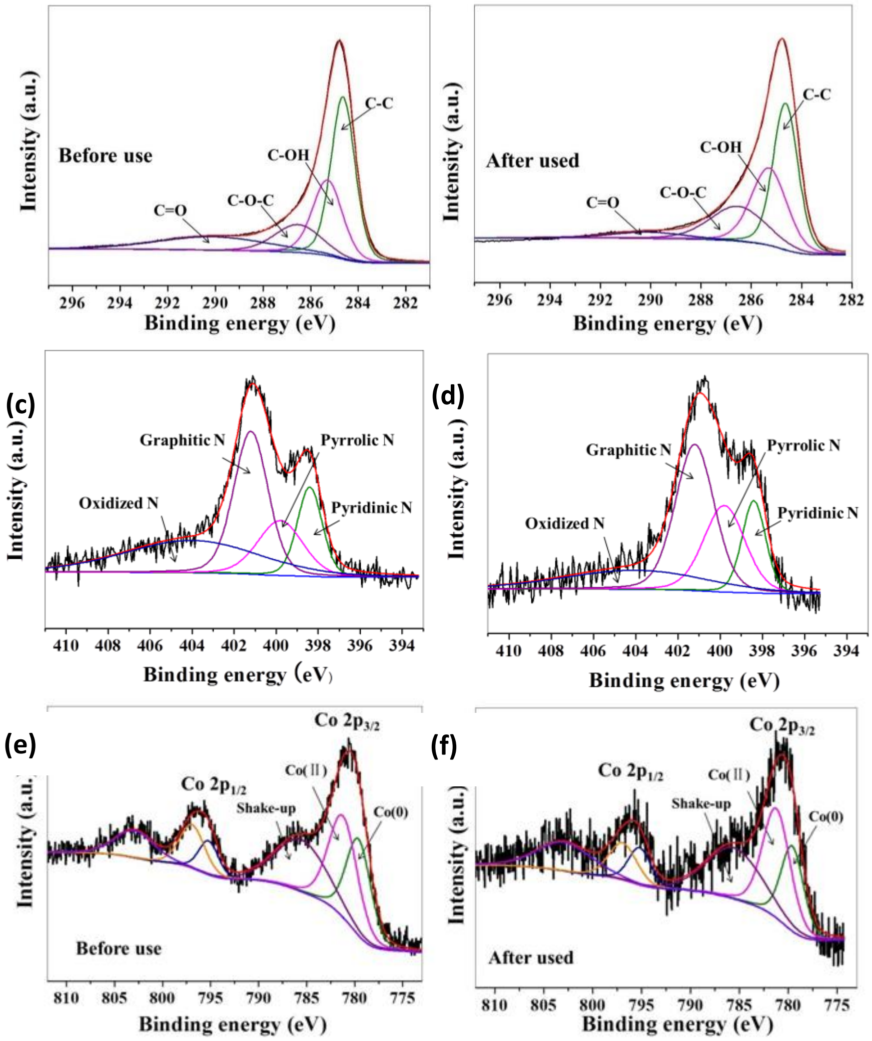

Figure S8. XPS C 1s, N 1s and Co 2p core-level spectra of NRGO@Co hybrid before and after used.
